# *Aquimarina* sp. Associated With a Cuticular Disease of Cultured Larval Palinurid and Scyllarid Lobsters

**DOI:** 10.3389/fmicb.2020.573588

**Published:** 2020-10-09

**Authors:** Mei C. Ooi, Evan F. Goulden, Andrew J. Trotter, Gregory G. Smith, Andrew R. Bridle

**Affiliations:** ^1^ Institute for Marine and Antarctic Studies, University of Tasmania, Hobart, TAS, Australia; ^2^ Department of Agriculture and Fisheries, Bribie Island Research Centre, Woorim, QLD, Australia

**Keywords:** white leg disease, *Aquimarina* sp., Koch’s postulates, cultured lobster, Palinurid larvae, Scyllarid larvae

## Abstract

Shell (cuticular) disease manifests in various forms and affects many crustaceans, including lobsters. Outbreaks of white leg disease (WLD) with distinct signs of pereiopod tissue whitening and death have been observed in cultured larvae (phyllosomas) of ornate spiny lobster *Panulirus ornatus*, eastern rock lobster *Sagmariasus verreauxi*, and slipper lobster *Thenus australiensis*. This study aimed to characterise and identify the causative agent of WLD through morphological and molecular (16S rRNA gene and whole genome sequencing) analysis, experimental infection of damaged/undamaged *P. ornatus* and *T. australiensis* phyllosomas, and bacterial community analysis (16S rRNA gene amplicon sequencing) of *P. ornatus* phyllosomas presenting with WLD during an outbreak. Bacterial communities of WLD-affected pereiopods showed low bacterial diversity and dominant abundance of *Aquimarina* spp. compared to healthy pereiopods, which were more diverse and enriched with *Sulfitobacter* spp. 16S rRNA gene Sanger sequencing of cultures from disease outbreaks identified the dominant bacterial isolate (TRL1) as a Gram-negative, long non-flagellated rod with 100% sequence identity to *Aquimarina hainanensis*. *Aquimarina* sp. TRL1 was demonstrated through comparative genome analysis (99.99% OrthoANIu) as the bacterium reisolated from experimentally infected phyllosomas presenting with typical signs of WLD. Pereiopod damage was a major predisposing factor to WLD. Histopathological examination of WLD-affected pereiopods showed masses of internalised bacteria and loss of structural integrity, suggesting that *Aquimarina* sp. TRL1 could enter the circulatory system and cause death by septicaemia. *Aquimarina* sp. TRL1 appears to have important genomic traits (e.g., tissue-degrading enzymes, gliding motility, and aggregate-promoting factors) implicated in the pathogenicity of this bacterium. We have shown that *Aquimarina* sp. TRL1 is the aetiological agent of WLD in cultured Palinurid and Scyllarid phyllosomas and that damaged pereiopods are a predisposing factor to WLD.

## Introduction

Shell (cuticular) diseases of crustaceans are a global phenomenon with origins dating back more than a 100 million years (Klompmaker et al., 2016). Shell diseases typically present as “spots” that progress to necrotic lesions of varying severity on shell surfaces. Bacteria capable of degrading key cuticular biopolymers are frequently associated with these lesions (Vogan et al., 2008; Bell et al., 2012). Shell diseases affecting lobsters have been described in both wild populations and animals in post-capture holding facilities, and include epizootic shell disease (ESD) of American lobster *Homarus americanus* and tail fan necrosis of spiny (Palinurid) lobsters (reviewed by Shields, 2011). A number of behavioural and environmental drivers are hypothesised to facilitate cuticular disease, including abrasion and injury (Quinn et al., 2012), sea temperatures (Tlusty and Metzler, 2012), ocean acidification (Kunkel et al., 2012), and anthropogenic pollutants (Laufer et al., 2012). The most studied of lobster cuticular disease is ESD, which has spread along the East Coast of the United States, causing major losses in abundance, marketability, and economic value (Castro et al., 2012; Gomez-Chiarri and Cobb, 2012).

There is now an emerging view that many marine diseases including shell diseases are caused by dysbiosis of the healthy microbiome (Egan and Gardiner, 2016). Indeed, the current mechanistic view for ESD is that increased sea temperatures and anthropogenic contaminants suppress host defences and weaken the cuticle, driving dysbiosis of the cuticle bacteriome (Maynard et al., 2016). A number of studies on wild and cultured juvenile *H. americanus* have shown that bacterial communities of shell disease lesions harboured an increased abundance of certain strains, including *Aquimarina macrocephali* subsp. “*homaria*” when compared to healthy surfaces (Chistoserdov et al., 2012; Meres et al., 2012; Feinman et al., 2017), and conversely, when the strain was administered to abraded lobsters, it caused ESD-like lesions (Quinn et al., 2012). Another closely related bacterium, *Aquimarina hainanensis* was implicated in the mortalities in larvae of Pacific white shrimp *Litopenaeus vannamei* (Zheng et al., 2016) and mud crab *Scylla serrata* (Midorikawa et al., 2020). Chitinolytic virulence factors are common to bacteria that cause shell disease, and both *A. macrocephali* and *A. hainanensis* have demonstrated capacity for exoskeletal chitin degradation through chitin hydrolysis assays and analysis of genomes for chitinase-encoding genes (Ranson et al., 2018; Midorikawa et al., 2020).

Surveillance and diagnosis of emerging diseases (Goulden et al., 2012; Ooi et al., 2017) are required as aquaculture technologies for Palinurid and Scyllarid lobsters transition to commercial scale (Smith et al., 2017). Several disease conditions have been reported anecdotally within experimental hatcheries of Palinurid lobsters, ornate spiny lobster (*Panulirus ornatus*) and eastern rock lobster (*Sagmariasus verreauxi*), and the Scyllarid slipper lobster *Thenus australiensis* from around the Australasian region (pers. comm. the authors). One of the most devastating conditions affecting the planktonic larval (phyllosoma) stage is a cuticular affliction colloquially termed as white leg disease (WLD). WLD is characterised by signature whitening of the articulated appendages, including pereiopods, maxillipeds, antennae, and eyestalks. The infections typically progress from appendage extremities toward the cephalothoracic shield, which can result in: (1) loss of affected limb and incapacitation, (2) partial or total exuvial entrapment and mortality, or (3) infection of the cephalic shield, causing mortality 2–3 days after the initial signs of limb whitening. WLD outbreaks have been reported at experimental hatcheries in Western Australia, Queensland, and Tasmania in Australia, as well as in Tuaran, Malaysia. WLD was previously observed at UTAS during the culture of *S. verreauxi* and outbreaks have been observed consistently at the Institute for Marine and Antarctic Studies (IMAS) since the initial culture of *P. ornatus* (2010). Preliminary investigations by the authors into the causative agent of WLD using 16S rRNA analysis have putatively identified *Aquimarina hainanensis* as responsible for WLD outbreaks at IMAS and a geographically distant Queensland hatchery. Outbreaks of this disease continue to occur in *P. ornatus* as well as the more recent culture of *T. australiensis* (2017) at IMAS. The precise drivers for WLD remain unclear, although preliminary studies conducted by researchers at IMAS have implicated prevailing culture water chemistry and density-dependent factors. Under high-density culture it is likely that collisions and mechanical damage facilitate cuticular infections and horizontal transfer through proximal contact and/or cannibalism (pers. obs. the authors).

Industry uptake and expansion of the Palinurid and Scyllarid aquaculture sectors will be contingent on characterising new, emerging and exotic diseases that pose threats to productivity. This study aimed to identify the causative agent of WLD by (1) using 16S rRNA gene amplicon sequencing to compare bacterial communities of healthy and WLD-affected *P. ornatus* phyllosomas from an outbreak, (2) isolating and characterising the isolate through morphological and molecular (16S rRNA gene and whole genome sequencing) methods, and (3) fulfilling Koch’s postulates through experimental infection.

## Materials and Methods

### Bacterial Isolation

Bacterial communities were recovered from WLD outbreaks in *P. ornatus*, *T. australiensis*, and *S. verreauxi* experimental larviculture systems at IMAS, Hobart, Australia. All phyllosomas were collected individually into sterile seawater and placed on a sterile dissecting stage (Petri dish). To isolate culturable bacteria associated with WLD, individual WLD-affected pereiopods were removed aseptically by surgical blade and homogenised in sterile seawater. The homogenate was plated in triplicate on Zobell’s marine agar 2,216 (ZMA; Amyl Media, Australia) using spread plate method and incubated at 28°C for 48 h. Isolates were cultured to purity on ZMA at 28°C for 48 h and cryopreserved in marine broth (MB, Difco Laboratories, United States) with 30% (v/v) glycerol at −80°C. Isolates were revived in MB at 28°C with shaking (100 rpm) for assays. Samples used for 16S rRNA gene amplicon sequencing analysis of bacterial communities were from pereiopods of three healthy (no signs of WLD) and three WLD-affected *P. ornatus* phyllosomas removed as described and individually stored in 1 ml nucleic acid preservation solution (4 M ammonium sulphate, 25 mM sodium citrate, 10 mM EDTA, pH 5.2) at 4°C overnight followed by transfer to −20°C in preparation for further analysis (section “WLD-Associated Community Characterisation”).

### WLD-Associated Community Characterisation

Total nucleic acid was extracted from preserved pereiopod samples (section “Bacterial Isolation”) using a similar protocol to section “Sanger Sequencing” with the modification of extraction buffer composition (4 M Urea, 1% SDS, 0.2 M NaCl, and 1 mM sodium citrate) supplemented with Proteinase K. The V1–V3 hypervariable region of bacterial 16S rRNA was amplified using barcoded fusion primers with the same PCR reactions, thermal cycling program, and gel electrophoresis as described by Ooi et al. (2017). Barcoded amplicon sample libraries were sent to Macrogen (Seoul, Korea) for 16S rRNA gene amplicon sequencing using the 454 GS-FLX Titanium (Roche, United States) platform, and the sequences were deposited in NCBI Sequence Read Archive under BioProject accession number PRJNA659570.

The 16S rRNA gene amplicon sequencing files were demultiplexed according to barcodes, and primers were trimmed in Geneious (Kearse et al., 2012). The sequences were processed for 16S rRNA amplicon analysis using CloVR pipeline (White et al., 2011) from Data Intensive Academic Grid computational cloud (DIAG, 2016). Chimeric and poor quality sequences were removed using UCHIME and QIIME. Operational taxonomic units (OTUs) or clusters of filtered sequences with 95% nucleotide sequence identity were assigned to known taxa using the RDP Bayesian Classifier with a confidence threshold of 0.5. Files from CLoVR were uploaded to MicrobiomeAnalyst (Dhariwal et al., 2017) to examine sampling depth, alpha diversity, beta diversity, and OTU abundance. Data were filtered by removing OTUs (minimum two counts) with ≤10% prevalence and normalised by rarefying to the minimum library size, i.e., 4,881. MetagenomeSeq (fit feature model) compared OTU abundance (mean ± standard error) between two health statuses, and a false discovery rate-adjusted value of *p* < 0.05 was considered significant. A normality test was conducted before comparing each alpha diversity index between phyllosomas of different health statuses using independent samples *t*-test in SPSS v20, and considered significant when *p* < 0.05.

### Bacterial Characterisation

#### Sanger Sequencing

Marine broth cultures of the predominant colony morphotype were centrifuged at 10,000 *g*. Total nucleic acid (TNA) was extracted from pellets in 300 μl of extraction buffer (4 M urea, 0.5% SDS, 50 mM TRIS, and 10 mM EDTA) at 55°C for 10 min. Protein was removed by precipitation using half of the total volume of 7.5 M ammonium acetate (Sigma-Aldrich, Australia). Nucleic acid was precipitated from the supernatant with an equal volume of isopropanol with 0.2 μg μl^−1^ pink co-precipitant (Bioline, Australia) and centrifuged at 16,000 *g* for 10 min. The nucleic acid pellet was rinsed twice with 60% ethanol and resuspended in 40 μl LC-MS grade water (LiChrosolv, Merck, Australia). TNA extracts were subject to PCR amplification of the bacterial 16S rRNA gene. PCR reaction mixtures (10 μl) comprised 10 μl of 2 × MyTaq HS mix (Bioline), 400 nM each of 27F (5'-AGAGTTTGATCMTGGCTCAG-3') and 1492R (5'-TACGGYTACCTTGTTACGACTT-3') 16S rRNA gene primers and 2 μl of 1:10 diluted TNA. The PCR was conducted using a C1000 Thermal Cycler (Bio-Rad Laboratories, Australia) with the following thermal cycling program: initial denaturation for 3 min at 95°C, 28 cycles of denaturation for 15 s at 95°C, annealing for 30 s at 55°C, extension for 30 s at 72°C, and a final extension for 3 min at 72°C. PCR amplicons were purified using SureClean (Bioline) according to manufacturer’s instructions, quantified using a Qubit fluorometer (Invitrogen, Life Technologies Australia). Samples were sent to the Australian Genome Research Facility (AGRF) for Sanger sequencing using the same primers as the amplification reactions. Sequences were edited using Geneious 8.1.7 software and identified using NCBI Basic Local Alignment Search Tool (BLAST).

#### Whole Genome Amplification (WGA)

Whole genome amplification was performed using a 2-day old MB cultures of original (section “Bacterial Isolation”) and reisolated *Aquimarina* sp. (section “Fulfilment of Koch’s Postulates”). Bacterial TNA was extracted as in section “Sanger Sequencing” with modifications to the extraction buffer composition (4 M urea, 0.5% SDS, 0.2 M NaCl, and 10% glycerol) and addition of Proteinase K (Bioline). RNA was digested using 1 μl of RNase A (Thermo Fisher Scientific, Australia) with 30 min incubation at 37°C and gDNA precipitated then resuspended in buffered water (10 mM TRIS, 0.025% Triton X-100). Genomic DNA was assessed by 0.5% agarose gel electrophoresis for high molecular weight DNA and absence of RNA, followed by quantification using a Qubit fluorometer. Samples were sequenced using 150 bp paired-end reads on a MiSeq sequencer (Illumina, United States) by AGRF, while long reads were obtained using the Rapid Barcoding Sequencing Kit (SQK-RBK004, Oxford Nanopore Technologies, United Kingdom) and a R9.4.1 flow cell in the portable MinION Mk1B nanopore sequencer. Long-read nanopore sequences produced by the MinION (FAST5 files from MinKNOW) were basecalled in high accuracy mode, filtered with minimum q score 7, and trimmed of barcodes using Guppy version 3.5.2 (configuration file dna_r9.4.1_450bps_hac.cfg).

The genomes of the original and reisolated *Aquimarina* sp. were assembled *de novo* from Illumina Miseq and long-read MinION sequences using the Unicycler hybrid assembly pipeline 0.4.8.0 (Wick et al., 2017) in Galaxy version 20.01 (Afgan et al., 2018). The quality of assembled genomes was assessed using the Quality Assessment Tool for Genome Assemblies 5.0.2 (QUAST; Gurevich et al., 2013) for completeness using BUSCO version 3.0.2 with the bacteria_odb9 lineage (Seppey et al., 2019) and assembly graphs visualized using Bandage Image 0.8.1 (Wick et al., 2015). The species identities of the isolates were assessed from the assembled genomes using TrueBacID (Ha et al., 2019) and genes were annotated using the Rapid Annotation and Subsystem Technology (RAST) server (Aziz et al., 2008). A circular plot showing BLAST comparison of the *Aquimarina* sp. genomes was generated using BLAST Ring Image Generator (BRIG) version 0.95 (Alikhan et al., 2011) that incorporates NCBI nucleotide BLAST 2.10.1+. A genomic comparison between the initial and reisolated *Aquimarina* sp. was made using OrthoANI (Lee et al., 2016). The genome of original isolate *Aquimarina* sp. TRL1 was deposited in GenBank under the accession numbers CP053590 (chromosome) ‐ CP053591 (plasmid).

#### Morphology

*Aquimarina* sp. TRL1 MB cultures were heat fixed and Gram stained using standard methods (Smith and Hussey, 2005). Cell morphology was determined by transmission electron microscopy. Briefly, 1-day old MB cultures were vortexed for 3 s and deposited on a Formvar/carbon grid, negatively stained with 0.5% uranyl acetate, and examined with an electron microscope (Hitachi HT7700, Japan) at 80 kV.

### Experimental Infection

#### Inoculum Preparation

*Aquimarina* sp. TRL1 MB cultures were washed by centrifugation (5 min at 4,500 *g*, RT) and resuspended in autoclaved artificial seawater (Instant Ocean, Aquarium Systems, France). Bacterial concentrations of inoculum preparations were estimated by a direct count using a Neubauer hemocytometer. Bacterial concentrations represented as CFU ml^−1^ were assessed by a drop plate method using 10 μl drops of 10-fold serial dilutions of inoculum (Herigstad et al., 2001).

#### Aquarium

The pathogenicity of *Aquimarina* sp. TRL1 toward phyllosomas of *P. ornatus* [stages 6–10, 86 days post hatch (dph)] and *T. australiensis* (stage 3, 12 dph) was examined by immersion challenge. Healthy phyllosomas without signs of WLD were quarantined and transferred to a biosecure PC2 aquatic facility for experimental manipulations (IMAS, Hobart, Australia). Phyllosomas (*n* = 4 per treatment group) were immersed either intact or with a single surgically damaged pereiopod in glass beakers containing autoclaved seawater (control) or autoclaved seawater with low (2.7 × 10^5^ CFU ml^−1^ for *P. ornatus*; 8 × 10^6^ CFU ml^−1^ for *T. australiensis*) or high (9.0 × 10^7^ CFU ml^−1^ for *P. ornatus*; 4.5 × 10^8^ CFU ml^−1^ for *T. australiensis*) concentrations of *Aquimarina* sp. TRL1 for 30 min. Phyllosomas were transferred to 5 L kreisel aquaria at a stocking density of 0.8 larva L^−1^. Flow through, ozonated, and UV-irradiated seawater was supplied at two exchanges h^−1^. The photoperiod was set at 12:12 h light:dark. Water quality was measured daily, and parameters were within the following ranges: temperature 26.1–29.8°C, pH 7.65–8.23, salinity 34.2–35.9 ppt, and dissolved oxygen 7.97–10.06 mg l^−1^ for *P. ornatus* systems; and temperature 27.1–29.6°C, pH 7.90–8.15, salinity 33.1–34.8 ppt, and dissolved oxygen 7.85–8.58 mg l^−1^ for *T. australiensis* systems. Animals were not fed during the experiment. Phyllosomas were evaluated daily for signs of WLD and mortality. Upon termination of the experiments at day 3 (*P. ornatus*) or day 4 (*T. australiensis*), phyllosomas were collected for reisolation of the pathogen and histopathological examination.

#### Fulfilment of Koch’s Postulates

Pereiopods of all phyllosomas were surgically removed and homogenised in autoclaved artificial seawater. Homogenates were spread plated on ZMA and incubated at 28°C for 1–4 days. Three dominant colony morphotypes from each homogenate were cultured to purity in MB. A real-time PCR that amplified a 156 bp amplicon of the 16S rRNA gene of *Aquimarina* spp. was used to screen pure MB cultures for representative isolates for sequencing. The PCR primer set was designed using Beacon Designer 8.10 (Premier Biosoft, United States). The specificity of the primer set was tested *in silico* against the NBCI nr nucleotide database and found to amplify *Aquimarina* spp. and closely related members of *Flavobacteriaceae*. Real-time PCR reaction mixture (10 μl) consisted of 5 μl of 2 × MyTaq HS mix with SYBR Green, 200 nM each of *Aquimarina* F1 (5'-CCTTACCAGGGCTTAAATGT-3') and R1 (5'-AACCTGCTAGCAACTAACAA-3') primers and 0.2 μl of bacterial culture. The real-time PCR was conducted using CFX Connect Real-Time System (Bio-Rad Laboratories) with the following thermal cycling program: 3 min at 95°C, 40 cycles of 10 s at 95°C, 20 s at 58°C, and 10 s at 72°C. Specificity of amplicons was examined using melt curve analysis performed at 95°C for 10 s, 58°C for 5 s, and gradually increased to 95°C. The positive isolates were subject to Sanger and whole genome sequencing.

#### Histology

Pereiopods of control (no lesions) and *Aquimarina* sp. TRL1-exposed (lesions) phyllosomas were fixed in seawater Davidson’s fixative (3:3:2:1 seawater:ethanol:formalin:acetic acid) for 24–48 h at room temperature before transferring to 70% ethanol. Fixed pereiopods were processed in a Leica TP1050 tissue processor (Leica Biosystems, Australia). Samples were embedded in paraffin using a Shandon Histocentre 3 embedding center (Thermo Electron Australia). Samples were sagitally sectioned at 4 μm using a Microm HM340 rotary microtome (Microm International, Germany) and stained with haematoxylin and eosin using a Shandon Linistain GLX automatic stainer (Thermo Fisher Scientific). Sections were examined using a Leica DM500 light microscope with images being captured using a Leica ICC50 W camera and processed using LAS EZ software (Leica Microsystems, Australia).

## Results

### Bacterial Communities Associated With WLD

**Figure 1:**
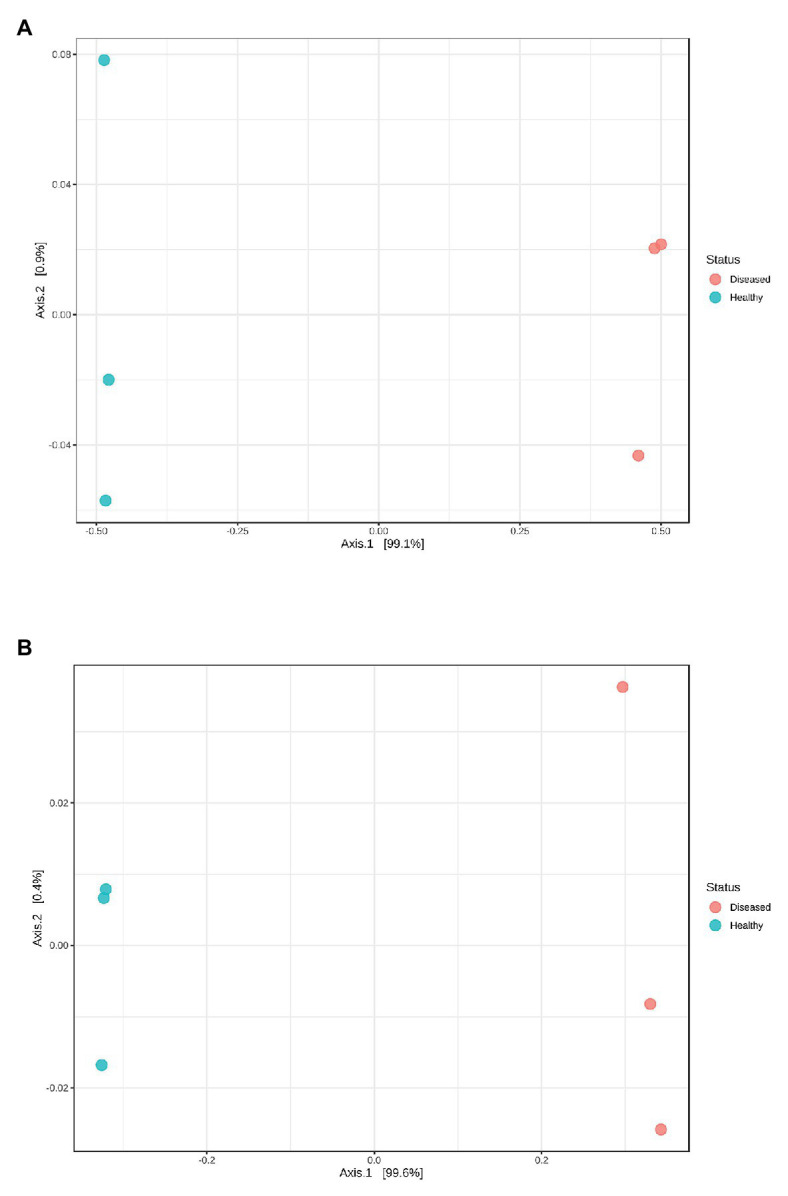
Principal coordinate analysis plots based on **(A)** Bray Curtis index and **(B)** weighted UniFrac distance methods showing similarity in pereiopod sequence libraries of *P. ornatus* phyllosomas.

The mean read length of 16S rRNA gene amplicon sequences was 486 bp. Good’s coverage ranged between 99.8 and 100% (Table 1). The observed OTUs (*t*_4_ = −5.594, *p* = 0.013) and ACE indices (*t*_4_ = −3.892, *p* = 0.042) were significantly lower in the diseased phyllosomas compared to the healthy animals (Table 1). This was consistent with the diseased phyllosomas library being significantly less diverse than the healthy animals according to the Shannon (*t*_4_ = −4.383, *p* = 0.012) and Simpson indices (*t*_4_ = −3.474, *p* = 0.028). The first axes of principal coordinate analyses based on Bray Curtis and weighted UniFrac distance matrices explained 99.1 and 99.6% of the OTUs variation between sample libraries (Figure 1). Both sets of PCoA analyses showed bacterial communities from healthy animals clustered distinctly from WLD-affected communities.

**Table 1:** Sampling depth, richness, and alpha diversity indices for pereiopod sequence libraries of *P. ornatus* phyllosomas.

Sample ID	Sampling depth			Richness estimators		Diversity indices	
	Filtered sequences	Observed OTUs^*^	Good’s coverage (%)	Chao1	ACE^*^	Shannon^*^	Simpson^*^
Healthy 1	8,302	51	99.8	64	60	0.91	0.31
Healthy 2	9,217	39	99.9	40	40	0.90	0.35
Healthy 3	8,851	39	99.9	41	41	0.67	0.22
Diseased 1	5,039	16	100.0	16	16	0.25	0.08
Diseased 2	5,202	17	99.9	19	22	0.50	0.18
Diseased 3	4,881	22	99.9	24	24	0.33	0.11

* significantly different indices between health status (*p* < 0.05).

**Figure 2:**
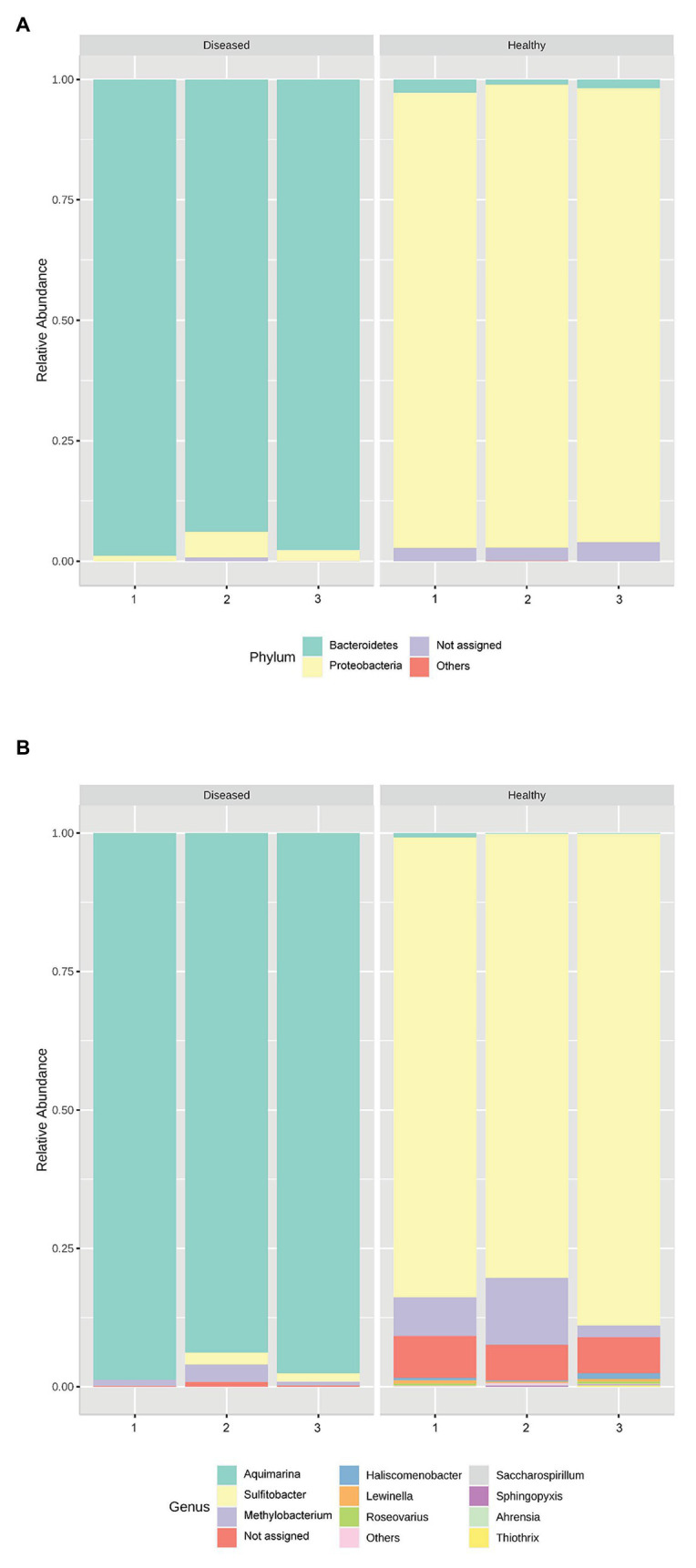
Relative abundance of the bacterial **(A)** phyla and **(B)** genera in the pereiopods of *P. ornatus* phyllosomas (*n* = 3 per treatment group).

Genus *Aquimarina* (phylum Bacteroidetes) was significantly more represented (*p* < 0.001) in the pereiopod of diseased *P. ornatus* phyllosomas (96.7 ± 1.5%) than that of healthy larvae (0.4 ± 0.2%; Figure 2). Conversely, the pereiopod libraries of healthy phyllosomas had significantly higher abundance of genera *Sulfitobacter* (phylum Proteobacteria; 84.0 ± 2.5%; *p* < 0.001) and *Methylobacterium* (phylum Proteobacteria; 7.1 ± 2.9%; *p* = 0.033) than that of diseased animals (*Sulfitobacter* 1.2 ± 0.6%; *Methylobacterium* 1.7 ± 0.8%).

### Characterisation of *Aquimarina* sp. TRL1

#### WGA and Identification

**Figure 3:**
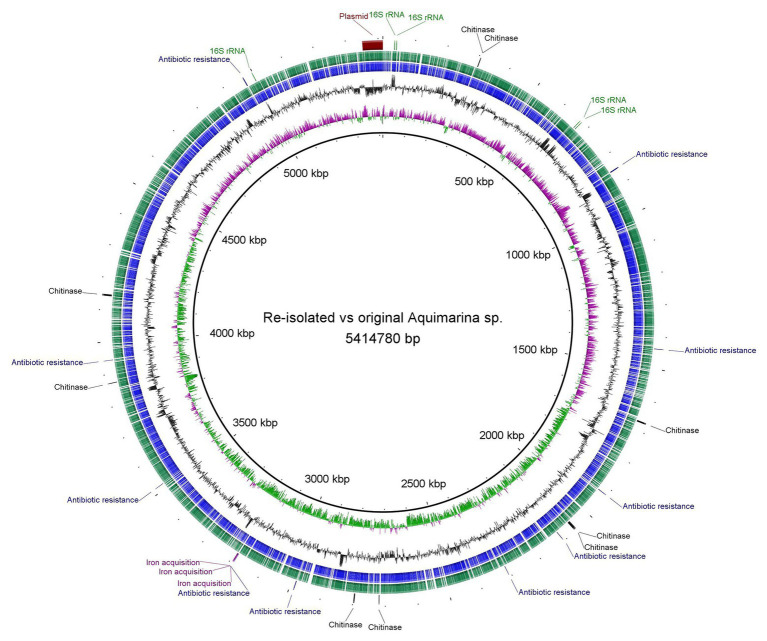
Circular representation and Basic Local Alignment Search Tool (BLAST) comparison of the isolated *Aquimarina* sp. genomes analysed and generated using BLAST Ring Image Generator (BRIG). Circular tracks display (from the inside): (1) GC skew, (2) GC plot, (3) coding sequences of original *Aquimarina* sp. TRL1, (4) coding sequences of reisolated *Aquimarina* sp., and (5) encoded genes (16S rRNA, chitinase, antibiotic resistance and iron acquisition) in the chromosome and the plasmid region.

Whole genome assembly of the original *Aquimarina* sp. TRL1 isolate (section “Bacterial Isolation”) using short (1.11 Gb, 3,676,944 paired-end reads) and long-read nanopore (254.3 Mb, 109,909 reads, mean read length = 2,313.4 bases, read length N50 = 8,572 bases) sequences with the Unicycler hybrid assembler and assessed by QUAST provided a single circular contig representing the 5,351,143 bp circular chromosome and a separate 63,637 bp circular plasmid (Figure 3). As a measure of genome completeness, the analysis of single-copy orthologs using BUSCO showed 93.9% complete and single BUSCOs. Annotation of the *Aquimarina* sp. TRL1 genomes using the RAST server identified genes associated with tissue-degrading enzymes (including eight chitinase genes), physical attributes, stress resistance, iron uptake, and antibiotic resistance that are potentially related to its virulence factors (Supplementary Table 1). Additionally, five 16S rRNA genes were detected. The 16S rRNA gene sequences obtained from the WGA (one isolate from *P. ornatus*) or from Sanger sequencing (three isolates from each of *P. ornatus*, *T. australiensis*, and *S. verreauxi*) from WLD outbreaks were a 100% BLAST match to *Aquimarina hainanensis* (GenBank accession KP200684). However, as the genome for type strain *A. hainanensis* (Zheng et al., 2016) was unavailable, *Aquimarina* sp. TRL1 could not be definitively classified as *A. hainanensis* according to the proposed minimal standards for the use of genome data for the taxonomy of prokaryotes (Chun et al., 2018). Nevertheless, the *Aquimarina* sp. TRL1 genome had 83.0% average nucleotide identity (ANI) when compared with the pathogen associated with ESD, *A. macrocephali*.

#### Morphology

**Figure 4:**
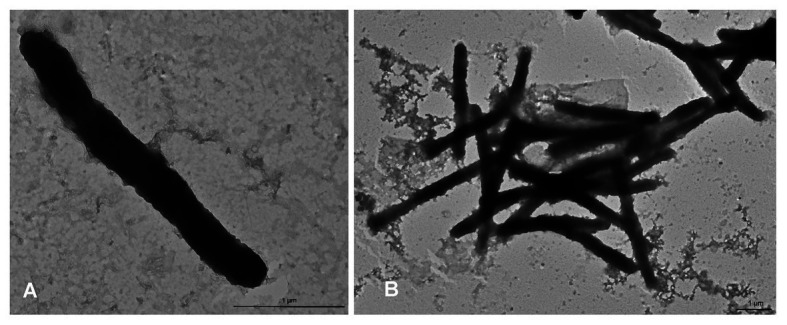
Transmission electron micrographs of *Aquimarina* sp. TRL1 from 1 d old marine broth cultures showing **(A)** an individual rod-shaped cell and **(B)** a mesh of cells.

The colony morphotype was odorous, yellow with convex round (~1 mm Ø) to rhizoid morphology after prolonged incubation. *Aquimarina* sp. TRL1 was a Gram-negative bacterium with non-flagellated, rod-shaped cells measuring 2.3–4.3 μm long and 0.3–0.4 μm wide (Figure 4). The cells typically formed mesh-like aggregates (Figure 4B), macroscopically observed as dense yellow agglomerates in broth cultures.

### Experimental Infection and Fulfilment of Koch’s Postulates

**Figure 5:**
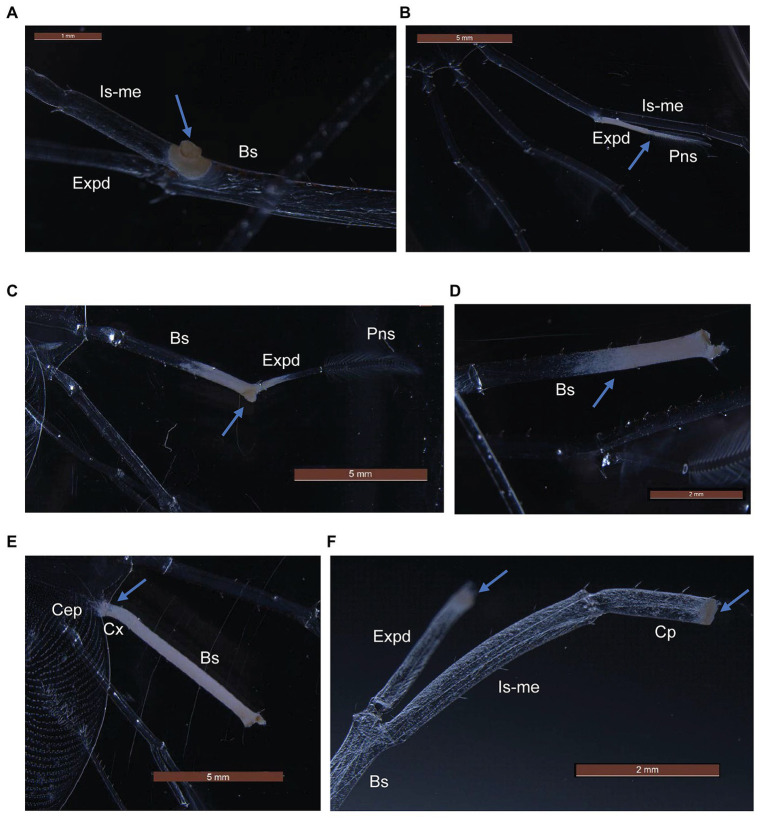
Gross pathological changes (arrows) during white leg disease (WLD) progression of *P. ornatus* **(A–E)** and *T. australiensis* **(F)** phyllosomas. **(A)** Pereiopod spot lesion at junction of basis (Bs), ischio-merus (Is-me) and exopod (Expd). **(B)** Advanced lesion of exopod and plumose natatory setae (Pns). **(C)** Detachment of pereiopod at junction of basis and ischio-merus. **(D)** Detachment of pereiopod and exopod at basis joint. **(E)** Creeping tissue whitening of pereiopod basis and coxa (Cx) segments and cephalic shield (Cep). **(F)** Spot lesions on carpus (Cp) and exopod of surgically damaged pereiopod (*T. australiensis*).

*P. ornatus* and *T. australiensis* phyllosomas exposed to high concentrations of *Aquimarina* sp. TRL1 exhibited white-yellow spot lesions on pereiopods as early as 1 day post-exposure. Lesions typically developed on surgically damaged pereiopods or one to two pereiopods of intact phyllosomas. The incidence of lesions was associated with the concentration of *Aquimarina* sp. TRL1, with more lesions observed in phyllosomas exposed to high concentrations. Necrotic spot lesions (Figures 5A,F) gradually progressed over 1–2 days to whitening of adjacent tissues (Figure 5B) in both species. For *P. ornatus*, this proceeded to detachment of segments or exopods (swimmeret) at the junction of the basis (Figure 5D) and ischio-merus (Figure 5C). *P. ornatus* phyllosomas also “curled” as the disease progressed, defined by the clustering of pereiopods and distortion of the cephalic shield. Moribundity and death was preceded by extensive and systemic tissue whitening of appendages and cephalic shield (Figure 5E). WLD lesions were not observed for intact or surgically damaged control phyllosomas of *P. ornatus* or *T. australiensis*.

**Figure 6:**
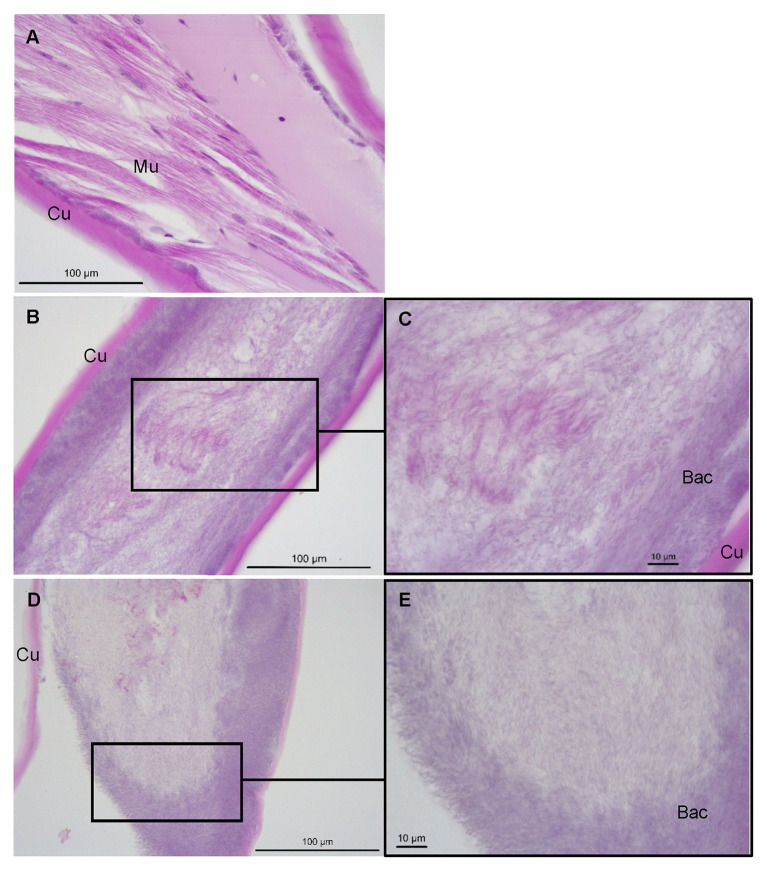
Sagittal sections of *P. ornatus* pereiopod muscle (Mu) and cuticular (Cu) architecture from **(A)** control phyllosomas and **(B–E)** phyllosomas exhibiting lesions following exposure to *Aquimarina* sp. TRL1. Bac: bacterial colonies. Sections were stained with haematoxylin and eosin.

Histological examinations of control *P. ornatus* showed structural integrity, with multinucleated muscle fibers and connective tissue underlying an intact cuticle (Figure 6A). Conversely, pereiopods with lesions from phyllosomas exposed to *Aquimarina* sp. TRL1 exhibited loss of muscle and cuticular architecture associated with masses of basophilic bacterial aggregations, which were denser at cuticular margins (Figures 6B–E).

On the 3rd day of experimental infection, cumulative mortality was 50 and 75% for surgically damaged *P. ornatus* phyllosomas exposed to low or high concentrations of *Aquimarina* sp. TRL1, respectively (Table 2A). No mortality was observed for intact *P. ornatus* phyllosomas exposed to *Aquimarina* sp. TRL1 at low concentration; however, 75% mortality was observed for intact phyllosomas exposed to the high concentration. On the 4th day of experimental infection, mortality (50%) of *T. australiensis* phyllosomas occurred only in surgically damaged animals exposed to the high concentration of *Aquimarina* sp. TRL1 (Table 2B). There was no mortality in intact or surgically damaged control phyllosomas of *P. ornatus* or *T. australiensis*.

**Table 2:** Cumulative mortality (%) of intact or surgically damaged **(A)** *P. ornatus* and **(B)** *T. australiensis* phyllosomas (*n* = 4 per treatment group) exposed to low or high concentrations of *Aquimarina* sp. TRL1.

A						
Concentration	Pereiopod	Cumulative mortality (%) on day				
		0	1	2	3	
Control	Intact	0	0	0	0	
	Damaged	0	0	0	0	
Low	Intact	0	0	0	0	
(2.7 × 10^5^ CFU ml^−1^)	Damaged	0	0	0	50	
High	Intact	0	25	50	75	
(9.0 × 10^7^ CFU ml^−1^)	Damaged	0	0	0	75	
B						
Concentration	Pereiopod	Cumulative mortality (%) on day				
		0	1	2	3	4
Control	Intact	0	0	0	0	0
	Damaged	0	0	0	0	0
Low	Intact	0	0	0	0	0
(8.0 × 10^6^ CFU ml^−1^)	Damaged	0	0	0	0	0
High	Intact	0	0	0	0	0
(4.5 × 10^8^ CFU ml^−^1)	Damaged	0	0	0	0	50

For *Aquimarina* spp. real-time PCR-positive amplification products were recovered from phyllosomas of both species inoculated with the *Aquimarina* sp. TRL1 isolate. The culturable pereiopod communities from control *P. ornatus* and *T. australiensis* were PCR negative. The Sanger sequenced 16S rRNA PCR amplicons from the PCR-positive isolates from *P. ornatus* and *T. australiensis* shared 98.1–100% sequence similarity with *Aquimarina hainanensis* (KP200684). The complete genome of the reisolated *Aquimarina* sp. (section “Fulfilment of Koch’s Postulates”) from *P. ornatus* assembled using short (1.42 Gb, 4,686,658 paired-end reads) and nanopore long reads (244.7 Mb, 125,272 reads, mean read length = 1,935.2 bases, read length N50 = 6,449 bases) consisted of a 5,341,386 bp circular chromosome and a 63,637 bp circular plasmid that had 93.9% complete and single BUSCOs. The genomes of original (TRL1) and reisolated *Aquimarina* sp. (Figure 3) were highly similar by BLAST (>99%) with a 99.99% OrthoANIu value. The high nucleotide similarities between the reisolated and original *Aquimarina* sp. suggest that TRL1 had been reisolated and together with the typical signs of WLD lesions, rapid progression of disease and acute mortality exhibited by experimentally infected *P. ornatus* and *T. australiensis* phyllosomas, Koch’s postulates were fulfilled.

## Discussion

The experimental infection in *P. ornatus* and *T. australiensis* confirmed the causative agent for WLD in cultured phyllosomas as *Aquimarina* sp. TRL1 through fulfilment of Koch’s postulates (Fredericks and Relman, 1996), whereby: (1) *Aquimarina* sp. TRL1 was present in diseased experimentally infected phyllosomas but absent in healthy control animals, (2) *Aquimarina* sp. TRL1 was successfully isolated from phyllosomas presenting with WLD and cultured to purity, (3) *Aquimarina* sp. TRL1 caused signs of WLD when introduced to healthy phyllosomas, and (4) *Aquimarina* sp. TRL1 was reisolated from experimentally infected lobsters presenting with WLD, as confirmed by Sanger and whole genome sequencing. Furthermore, *A. hainanensis* was isolated from *S. verreauxi* phyllosomas during a WLD outbreak. The identification of specific pathogens is critical to the development of rapid detection methods and surveillance, and in the notification of the broader aquaculture community of emergent issues (Stentiford et al., 2017).

*Aquimarina* sp. TRL1 is Gram-negative, yellow pigmented and non-flagellated bacterium typical of *A. hainanensis* strains (Zheng et al., 2016; Midorikawa et al., 2020) and other members of the *Aquimarina* genus (Yoon et al., 2011; Lin et al., 2012; Yu et al., 2013; Wang et al., 2018). The number of species assigned to the *Aquimarina* genus has been rapidly expanding in recent times, with many recovered from seawater including *A. addita* (Yi and Chun, 2011), *A. aggregata* (Wang et al., 2016), *A. atlantica* (Li et al., 2014a), *A. celericrescens* (Wang et al., 2018), *A. litoralis* (Oh et al., 2010), *A. longa* (Yu et al., 2013), *A. megaterium* (Yu et al., 2014), *A. muelleri* (Nedashkovskaya et al., 2005), and *A. pacifica* (Zhang et al., 2014) and marine organisms such as *A. agarilytica* and *A. agarivorans* from red alga (Lin et al., 2012; Zhou et al., 2015); *A. hainanensis* and *A. penaei* from shrimp *L. vannamei* (Li et al., 2014b; Zheng et al., 2016); *A. gracilis* and *A. mytili* from mussel *Mytilus coruscus* (Park et al., 2012, 2013); and *A. spongiae* from sponge *Halichondria oshoro* (Yoon et al., 2011). In particular, *A. hainanensis* has been associated with shell diseases and mortality in a number of species of larval (Zheng et al., 2016; Midorikawa et al., 2020) and juvenile (Dan and Hamasaki, 2015) crustaceans, and this was confirmed by the findings of our study. Strains of *A. hainanensis* may be active over a range of temperature in cultured larvae of crustaceans, observed at 23°C for *S. verreauxi* (this study); 25°C for brine shrimp *Artemia franciscana*, freshwater shrimp *Caridina multidentata*, swimming crab *Portunus trituberculatus*, and mud crab *S. serrata* (Midorikawa et al., 2020); and 28°C for *P. ornatus*, *T. australiensis* (this study), and *L. vannamei* (Zheng et al., 2016). Moreover, susceptibility towards *A. hainanensis* appears to be species-dependent (Midorikawa et al., 2020) and was confirmed by observations of lower cumulative mortality in *T. australiensis* phyllosomas compared to *P. ornatus* in this study.

From the experimental infection, we found that *Aquimarina* sp. TRL1 and limb damage were important determinants of WLD. Firstly, WLD did not manifest in control phyllosomas in the absence of inoculated *Aquimarina* sp. TRL1, irrespective of intactness of pereiopods. Secondly, a low concentration of *Aquimarina* sp. TRL1 was able to induce WLD in *P. ornatus* phyllosomas with surgically damaged pereiopods. And thirdly, cumulative mortality was the same in *P. ornatus* phyllosomas, irrespective of intactness of pereiopods, at a high level of exposure to *Aquimarina* sp. TRL1. As it is unlikely that *Aquimarina* are present in larval rearing systems at the highest concentrations used in this study, together these results suggest broken appendages predispose phyllosomas to WLD. The fragile articulated limbs of phyllosomas are easily broken due to collisions, water turbulence, and handling, which leave the cuticle vulnerable to invasion by chitinolytic bacteria. *Aquimarina* sp. TRL1 is involved with the initiation of spot lesions of WLD that progress to tissue whitening and loss of pereiopod segments and function. This will likely impact swimming and foraging ability preceding starvation. However, death due to septicaemia and systemic infection as revealed by histopathology probably occurs well in advance given pelagic phyllosomas are inherently adapted to long periods of starvation (Smith et al., 2010).

The complete genome analysis of *Aquimarina* sp. TRL1 revealed numerous potential virulence factors including substrate-degrading enzymes, gliding, aggregate-forming ability, and antibiotic resistance. Multiple genes encoding chitinases were detected in *Aquimarina* sp. TRL1, and this feature is common among members of *Aquimarina* recovered from *S. serrata* (Midorikawa et al., 2020), *H. americanus* (Ranson et al., 2018), and seawater (Xu et al., 2015). Encoded genes for haemolysin, metalloprotease, and iron uptake mechanisms were also described as virulence factors for *Aquimarina* sp. associated with ESD in *H. americanus* (Ranson et al., 2018). The presence of genes encoding chitinases, lipases, and proteases suggests functions in the degradation of exoskeletal tissues, allowing *Aquimarina* sp. to breach epithelial barriers. Once *Aquimarina* sp. is internalised and pervades the circulatory system, the presence of superoxide dismutase genes similar to the fish pathogen *Tenacibaculum maritimum* (Pérez-Pascual et al., 2017) may enable protection from oxidative stress responses of host phagocytes. *Aquimarina* sp. TRL1 also encoded multiple transporter proteins involved in gliding motility, which is common throughout *Flavobacteriaceae* (Bernardet and Bowman, 2006). Gliding motility facilitates movement along surfaces and together with enzymatic degradation could account for the distinct “creeping” of tissue whitening of phyllosomas. The differences in the regulation of motility systems among strains could be a major determinant of pathogenicity (Hudson et al., 2019). Host attachment may also occur more rapidly in more virulent strains, as shown for *Flavobacterium psychrophilum* attached to the gills of rainbow trout *Oncorhynchus mykiss* (Nematollahi et al., 2003). The formation of mesh-like aggregations of *Aquimarina* sp. TRL1 likely facilitated attachment to phyllosoma appendages, as shown for larvae of *S. serrata* and *P. trituberculatus* larvae (Midorikawa et al., 2020). Genes encoding glycosyltransferase may secrete extracellular polysaccharides that help the formation of aggregates, as proposed for *A. longa* (Xu et al., 2015). The *Aquimarina* sp. TRL1 chromosome also revealed genes encoding resistance to beta-lactams, chloramphenicol, acriflavin, and polymyxin antibiotics. Therefore, aquaculture antibiotics, such as amoxicillin, penicillin, chloramphenicol (Liu et al., 2017), and acriflavin (Mohamed et al., 2000) are unlikely to be effective when treating *Aquimarina* sp. TRL1 infections.

In our study, the bacteriome of pereiopods from healthy *P. ornatus* phyllosomas collected from a larval rearing system was dominated by *Sulfitobacter* spp. These microorganisms are dominant members of wild *P. ornatus* phyllosomas with putative, yet to be ascertained roles in health (Payne et al., 2008). *Sulfitobacter* is common in the marine environment, able to metabolise sulphur compounds (Pujalte et al., 2014), and in interaction with phytoplankton produces bioactive metabolites that inhibit important aquaculture pathogens (Sharifah and Eguchi, 2012). Conversely, the bacteriome of WLD-affected pereiopods of *P. ornatus* phyllosomas collected from a larval rearing system was dominated by *Aquimarina* spp. The dominance of *Aquimarina* (96.7%) in WLD communities of *P. ornatus* is in stark contrast to *Aquimarina* populations (11.8%) involved in polymicrobial diseases, such as ESD in *H. americanus* (Quinn et al., 2012; Feinman et al., 2017). This suggests a more pivotal role for *Aquimarina* spp. in the aetiology of WLD, which supports our observations under experimental infection. Another possibility is that larvae are more susceptible to *Aquimarina* causing an acute WLD compared to a chronic ESD in older lobsters. Moreover, *Aquimarina* spp. have been found in low abundance in healthy organisms, including *H. americanus* (Meres et al., 2012) and red alga *Delisea pulchra* (Kumar et al., 2016), suggesting some species are capable of opportunistic infection under certain conditions. Dysbiosis may occur in the early stages of WLD, where opportunistic microorganisms like *Aquimarina* spp. are able to proliferate by outcompeting other microorganisms and overcoming host defences using genomic artillery (e.g., antibiotic resistance, iron uptake systems, and superoxide dismutase production) as described for *Aquimarina* sp. TRL1 in this study. Future work may examine the temporal and functional role of *Aquimarina* in WLD, and how microbial networks are impacted during the progression of the disease.

We have shown that *Aquimarina* sp. TRL1 is the aetiological agent of WLD in cultured Palinurid (*P. ornatus*) and Scyllarid (*T. australiensis*) larvae by fulfilling Koch’s postulates. Host species together with suboptimal culture condition is likely to influence the susceptibility of phyllosomas to WLD. Damaged pereiopods are a predisposing factor to WLD, particularly when phyllosomas are in a marine environment with a low concentration of *Aquimarina* sp. TRL1. *Aquimarina* sp. TRL1 appears to have important genomic traits (e.g., tissue-degrading enzymes, gliding motility, and aggregate-forming factors) involved in the pathogenicity and the distinct signs of tissue whitening in phyllosomas. The characterisation of WLD will allow the development of health management strategies for the emerging lobster aquaculture industry.

## Data Availability Statement

The datasets presented in this study can be found in online repositories. The names of the repository/repositories and accession number(s) can be found in the article/Supplementary Material.

## Author Contributions

AB, MO, AT, and GS conceived and designed the experiments. MO, AB, EG, and AT performed the experiments. MO and AB analysed the data. MO, AB, EG, AT, and GS wrote the paper. All authors contributed to the article and approved the submitted version.

## Disclaimer

The views expressed herein are those of the authors and are not necessarily those of the Australian Government or Australian Research Council.

### Conflict of Interest

The authors declare that the research was conducted in the absence of any commercial or financial relationships that could be construed as a potential conflict of interest.
